# Transgenic studies reveal the positive role of *LeEIL-1* in regulating shikonin biosynthesis in *Lithospermum erythrorhizon* hairy roots

**DOI:** 10.1186/s12870-016-0812-6

**Published:** 2016-05-26

**Authors:** Rongjun Fang, Ailan Zou, Hua Zhao, Fengyao Wu, Yu Zhu, Hu Zhao, Yonghui Liao, Ren-Jie Tang, Yanjun Pang, Rongwu Yang, Xiaoming Wang, Jinliang Qi, Guihua Lu, Yonghua Yang

**Affiliations:** State Key Laboratory of Pharmaceutical Biotechnology, NJU-NJFU Joint Institute of Plant Molecular Biology, School of Life Sciences, Nanjing University, Nanjing, 210046 People’s Republic of China; Jiangsu University of Science and Technology, Zhenjiang, 212003 People’s Republic of China; Co-Innovation Center for Sustainable Forestry in Southern China, Nanjing Forestry University, Nanjing, 210037 People’s Republic of China; Department of Plant and Microbial Biology, University of California, Berkeley, CA 94720 USA

**Keywords:** Ethylene, Hairy roots, *LeEIL-1*, Overexpression, RNAi, Shikonin

## Abstract

**Background:**

The phytohormone ethylene (ET) is a key signaling molecule for inducing the biosynthesis of shikonin and its derivatives, which are secondary metabolites in *Lithospermum erythrorhizon*. Although ETHYLENE INSENSITIVE3 (EIN3)/EIN3-like proteins (EILs) are crucial transcription factors in ET signal transduction pathway, the possible function of EIN3/EIL1 in shikonin biosynthesis remains unknown. In this study, by targeting *LeEIL-1* (*L. erythrorhizon* EIN3-like protein gene 1) at the expression level, we revealed the positive regulatory effect of *LeEIL-1* on shikonin formation.

**Results:**

The mRNA level of *LeEIL-1* was significantly up-regulated and down-regulated in the *LeEIL-1*-overexpressing hairy root lines and *LeEIL-1*-RNAi hairy root lines, respectively. Specifically, *LeEIL-1* overexpression resulted in increased transcript levels of the downstream gene of ET signal transduction pathway (*LeERF-1*) and a subset of genes for shikonin formation, excretion and/or transportation (*LePAL*, *LeC4H-2*, *Le4CL-1*, *HMGR*, *LePGT-1*, *LeDI-2*, and *LePS-2*), which was consistent with the enhanced shikonin contents in the *LeEIL-1-*overexpressing hairy root lines. Conversely, *LeEIL-1*-RNAi dramatically repressed the expression of the above genes and significantly reduced shikonin production.

**Conclusions:**

The results revealed that *LeEIL-1* is a positive regulator of the biosynthesis of shikonin and its derivatives in *L. erythrorhizon* hairy roots. Our findings gave new insights into the molecular regulatory mechanism of ET in shikonin biosynthesis. *LeEIL-1* could be a crucial target gene for the genetic engineering of shikonin biosynthesis*.*

**Electronic supplementary material:**

The online version of this article (doi:10.1186/s12870-016-0812-6) contains supplementary material, which is available to authorized users.

## Background

The roots of medicinal plant *Lithospermum erythrorhizon* can specifically accumulate shikonin and its derivatives which have significant anti-inflammatory, antitumor, and antimicrobial activities [1–4]. These red naphthoquinone pigments are also excellent natural dyes widely used in the industries of fabric, food and cosmetics [5, 6].

In the last decade, the cell or hairy root culture systems of *L. erythrorhizon* have been successfully used to produce these valuable compounds through a two-stage culture system, i.e., (1) *L. erythrorhizon* cells or hairy roots are cultured in B5 multiplication medium under light for fast amplification, and (2) transferred into M9 production medium to produce shikonin pigments in darkness [7–9]. This excellent system has become a promising tool to better understand the metabolism of shikonin pigments. Genes encoding pivotal enzymes or regulators for shikonin biosynthesis, excretion and/or transportation have also been cloned and characterized, such as the *L. erythrorhizon* phenylalanine ammonia-lyase gene (*LePAL*) [10], the *L. erythrorhizon* cinnamic acid 4-hydroxylase gene (*LeC4H*) [11], the 3-hydroxy-3-methylglutaryl-coenzyme A reductase gene (*HMGR*) [12], the *L. erythrorhizon* p-hydroxybenzoate:geranyltransferase gene (*LePGT*) [13–15], the *L. erythrorhizon* pigment callus-specific gene (*LePS-2*) [16], and the *L. erythrorhizon* dark-inducible gene (*LeDI-2*) [17, 18]. Moreover, several factors, such as light [8, 19, 20], mineral elements [21, 22], fungal elicitor [23], culture medium [9], nitric oxide [24], methyl jasmonate [25], and ET [26], have been described to be crucial regulators of shikonin biosynthesis.

ETHYLENE INSENSITIVE3 (EIN3)/EIN3-like proteins (EILs) are plant-specific transcription factors which regulate various ET responses [27–29]. EIN3/EIL1 is a key integration node between ET and other signals in the complex molecular signaling network [30]. By binding to specific promoter elements, EIN3/EIL1 activates or represses the expression of target genes responsible for ET signaling, and thus modulates multiple ET-related responses of plants, such as those for development, phenotype, and adaptation to environmental stresses [31–33]. However, the knowledge of the relationship between EIN3/EIL and plant secondary metabolism remains limited.

In the medicinal plant *L. erythrorhizon*, ET is an important regulator of shikonin biosynthesis [26]. Furthermore, an optimal concentration of endogenous ET was presumed to be pivotal for shikonin formation [34]. *LeEIL-1*, a homolog of *Arabidopsis EIN3*, has been isolated from *L. erythrorhizon* cells. It was speculated to be important for ET-regulated shikonin biosynthesis [35]. However, the function of *LeEIL-1* in shikonin biosynthesis at the molecular level remains unknown. Functional study on *LeEIL-1* could be useful for elucidating the relationship among *LeEIL-1*, ET, and shikonin production.

In this study, two transgenic strategies, overexpression and RNA interference (RNAi), were applied to induce *LeEIL-1*-overexpression and *LeEIL-1*-RNAi transgenic hairy roots. The relationship between the expression pattern of *LeEIL-1* and shikonin production was investigated to offer new insights into the understanding of the possible function of *LeEIL-1* in shikonin biosynthesis.

## Results

### Hairy root induction, cultivation, and identification

**Fig. 1:**
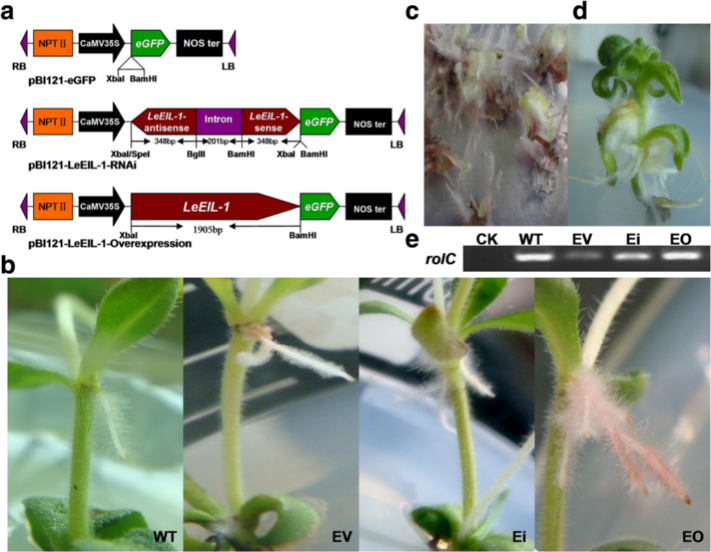
Induction, verification, and growth polymorphism observation of four different types of hairy root lines. (**a**) Structure and restriction maps of the transformation vectors pBI121-*eGFP* (EV), pBI121-*LeEIL-1*-RNAi (Ei), and pBI121-*LeEIL-1-*Overexpression (EO); (**b**) Growth polymorphism of the induced *L. erythrorhizon* hairy roots by using the method of pricking nodes with a needle. Differentiated callus, leaf (**c**), and regenerated shoots (**d**) from the Ei hairy root lines on the stock culture medium; (**e**) PCR analysis of the *rolC* gene in hairy roots. The untransformed *L. erythrorhizon* seedling was used as the negative control (CK)

For more detailed understanding of the function of *LeEIL-1* in shikonin formation, both RNAi and overexpression transgenic strategies were applied in this study. The plasmids of pBI121-*LeEIL-1-*Overexpression (EO) and pBI121-*LeEIL-1*-RNAi (Ei) were constructed and verified based on the pBI121-enhanced green fluorescent protein gene empty vector (pBI121-*eGFP*) (EV) (Fig. 1a; Additional file 1: Figure S1). The *Agrobacterium rhizogenes* strain 15834 (WT) and the *A. rhizogenes* 15834 containing EV, Ei or EO plasmid were employed to infect the nodes of aseptic seedlings [36]. Over 15 transgenic lines of hairy root had been successfully induced for each construct (i.e., EV, Ei, and EO) and WT (Fig. 1b).

For stock culture, all hairy roots (WT, EV, Ei, and EO) were transferred into B5 solid medium (hormone-free and antibiotics-free) at 26–28 °C under subdued light. The remarkable growth polymorphisms of four types of hairy roots were observed at the infection sites (Fig. 1b) or in the stock culture medium (Additional file 2: Figure S2). EO hairy root lines were displayed obviously red either at the infection sites of seedling nodes or in sub-cultured stock medium under subdued light. No obvious red hairy root lines were observed in WT, Ei, and EV. Many black segments were observed in most Ei. The growth rates of EO and Ei were relative slow in comparison with those of WT and EV in the stock culture medium or multiplication culture medium, with Ei exhibiting the slowest growth rate (Additional file 3: Figure S3). This finding indicated that the growth of *L. erythrorhizon* hairy roots might be affected by excessively low or high expression level of *LeEIL-1*. We also found that callusing and regeneration phenomena easily occurred in the Ei cultured in B5 medium (Fig. 1c and d) compared with other hairy root lines. Hence, we speculated that the repressed mRNA level of *LeEIL-1* changed the development and phenotype of hairy roots to some extent.

To confirm the transformation of hairy roots, the DNA samples from all transgenic hairy roots (WT, EV, Ei, and EO) were used as template for PCR amplification of the tagging *rolC* gene of *A. rhizogenes* 15834 [37, 38], and the DNA of untransformed *L. erythrorhizon* seedling was used as negative control. Results showed that the *rolC* gene was only amplified from WT, EV, Ei, and EO hairy roots, (Fig. 1e), which confirms the success of hairy root transformation of *L. erythrorhizon*.

**Fig. 2:**
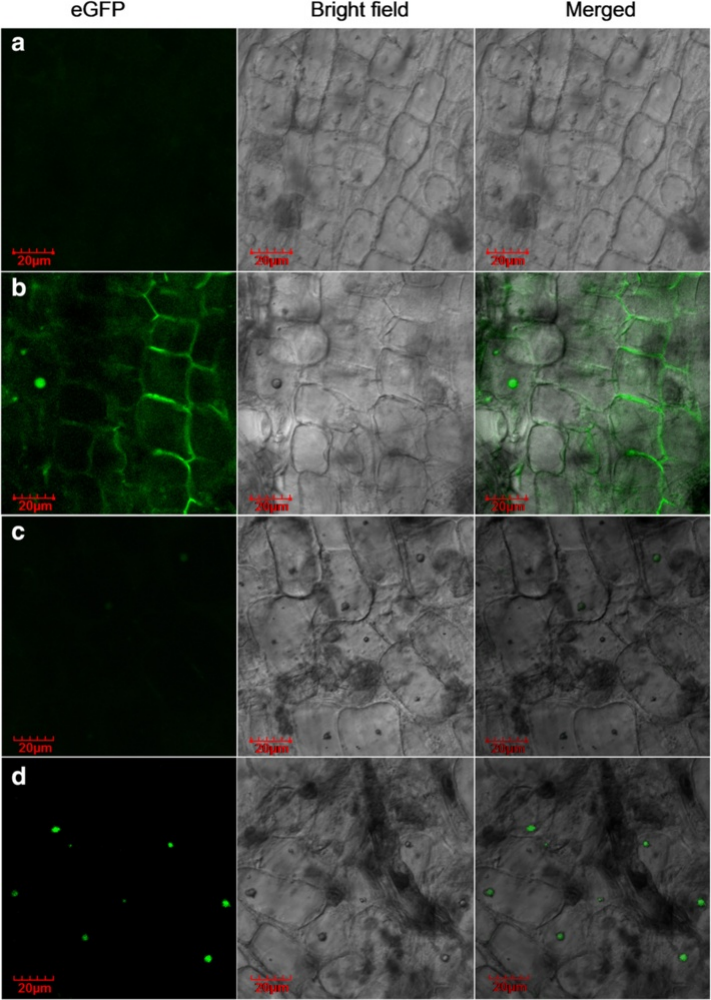
Confocal analyses of eGFP in WT (**a**), EV (**b**), Ei (**c**), and EO (**d**) hairy roots. *Scale bar* =20 μm; excitation wavelength = 488 nm; emission wavelength of eGFP = 510 nm

Confocal scanning laser microscopy analysis was performed on eGFP-tagged cells to identify all the hairy roots and clarify the subcellular localization of LeEIL-1. Different patterns of eGFP localization were visualized in hairy roots. No fluorescence could be observed in WT hairy roots (Fig. 2a), whereas uniform and intense signals of fluorescence appeared in the nucleus, cell wall and cytoplasm of EV hairy roots (Fig. 2b). In Ei hairy root lines, only weak fluorescence signal was discerned (Fig. 2c). In EO hairy root lines, strong signals emitted by eGFP were detected predominantly in the nucleus (Fig. 2d), where EIN3/EILs localized [39]. Based on these findings, we speculated that co-expression or co-localization of the fusion protein eGFP:LeEIL-1 may occur in the nucleus of EO but may be effectively suppressed in Ei hairy roots.

### Expression patterns of *LeEIL-1* in the two-stage culture system

**Fig. 3:**
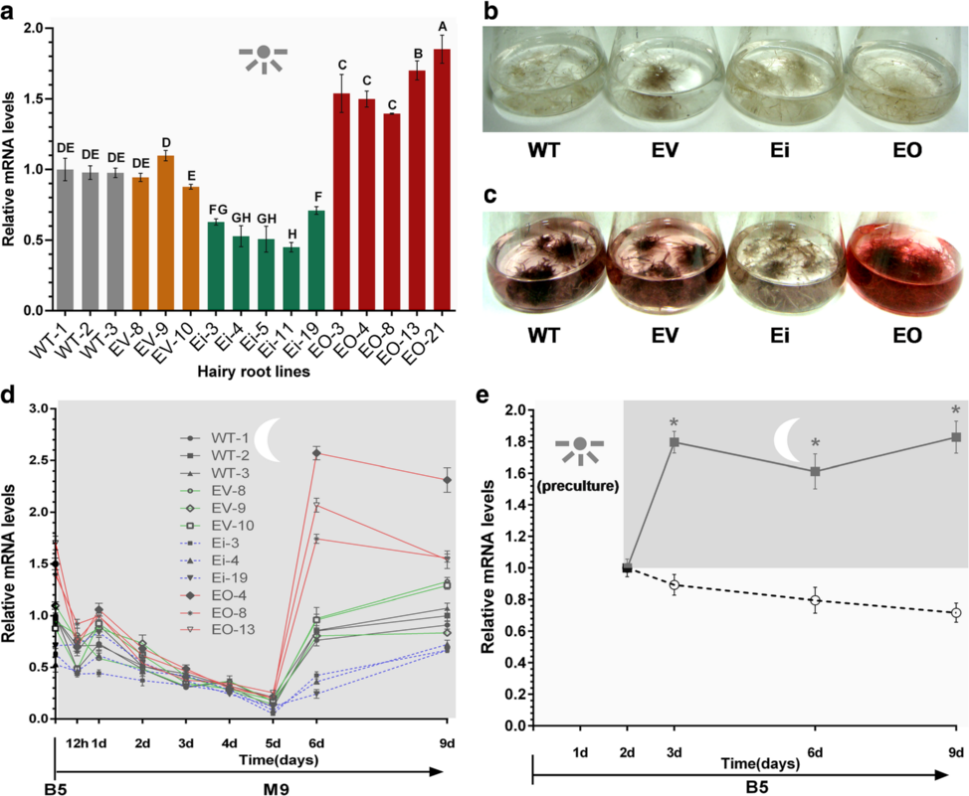
*LeEIL-1* Expression patterns and visual inspection of WT, EV, Ei, and EO hairy root lines. (**a**) Transcript levels of *LeEIL-1* in the randomly selected hairy root lines of WT, EV, Ei, and EO cultured in B5 medium under light at 26–28 °C for 15 days with constant shaking at 80 rpm. A representative example from two biological experiments was shown; data represent means ± SD (*n* = 3) and the *bars* with different *capital letters* indicate significant differences at *P <* 0.01 (Least Significant Difference); (**b**) Phenotypes of WT, EV, Ei, and EO hairy roots in conical flasks containing hormone-free B5 liquid medium for multiplication culture; (**c**) Visual inspection for the color changes of WT, EV, Ei, and EO hairy root lines in M9 for 6 days (20 ml medium/50 ml flask); (**d**) Dynamic expression pattern analysis of *LeEIL-1* when hairy root lines were transferred from B5 into M9 medium. The values of Ei and EO lines are significantly different from those of WT or EV lines at the time points of 6 or 9 days, respectively (Least Significant Difference, *P <* 0.05); (**e**) Expression patterns of *LeEIL-1* of the WT-1 during the dark/light transition, in which hairy roots were pre-cultured under light for 2 days and then transferred into darkness. The *asterisk* indicates that the mean value in the dark was significantly different from that under light at 3, 6 or 9 days time point, respectively (Student’s *t-*test, *P* < 0.05). The *sunshine logo* and *light gray* represent light period for B5 culture condition, and the *moon logo* and *deep gray* represent night period for M9 culture condition

In order to assess the transgenic effects, expression patterns of *LeEIL-1* in four types of hairy root were characterized by using real-time PCR method. Each five lines of Ei and EO cultured in B5 medium were randomly selected; each three lines of WT and EV were used as controls. Similar transcript levels were observed in three WT hairy root lines (WT-1, WT-2, and WT-3). Compared with that in WT hairy root lines, *LeEIL-1* did not significantly change in EV hairy root lines (*P* > 0.05). However, in Ei hairy root lines, *LeEIL-1* was significantly down-regulated compared with that in WT or EV (*P* < 0.01). In EO hairy root lines, *LeEIL-1* significantly increased (*P* < 0.01) (Fig. 3a). Therefore, the transcript level of *LeEIL-1* actually significantly increased in hairy roots of *LeEIL-1-*overexpressing and decreased in *LeEIL-1-*RNAi hairy root lines.

We speculated that the transformation of *LeEIL-1* genetically influences the biosynthesis of shikonin. Shikonin and its derivatives are red specific pigments, and color changes of the pigments in hairy roots as well as in M9 medium excreted from hairy roots were detected upon visual inspection [23]. Time-course analysis was also performed to assess shikonin pigment accumulation in hairy roots of WT, EV, Ei, and EO through visual inspection. No pigment was observed in B5 multiplication medium under light (Fig. 3b). The red color significantly changed when transferred hairy roots from B5 multiplication medium into M9 production medium for 1–6 days, reached the highest levels at 6–9 days, and exhibited minimal changes thereafter, with the highest yield found in EO and the lowest level in Ei from 1 to 9 days (Fig. 3c). The time course accumulation analyses of shikonin in different types of hairy roots were performed using the randomly selected four typical lines, i.e., WT-1, EV-9, Ei-19 and EO-13. From 1 to 6 days, the concentration of shikonin in hairy roots above increased rapidly, and then reached to a relative high level at the time point of 6 days. Thereafter, the content of shikonin in different hairy roots began to accumulate in different patterns. Significantly enhanced content of shikonin was observed in EO-13, while dramatically reduced in Ei-19 in comparison with that in EV-9 or WT-1 at each time point of 3, 6, 9 and 12 days (*P* < 0.05) (Additional file 4: Figure S4).

Based on the time-course analysis of shikonin formation, each three lines of WT, EV, Ei, and EO were selected and transferred from B5 multiplication medium into M9 production medium for the expression patterns analysis of *LeEIL-1*. Dynamic expression patterns of *LeEIL-1* in the M9 production medium (12 h and 1–9 days) were detected, similar to the expression pattern of *LeEIL-1* in *L. erythrorhizon* callus cells in our previous report [35]. Remarkable changes occurred when transferred hairy roots of WT, EV, Ei, and EO from B5 multiplication medium into M9 production medium for 9 days. The mRNA level of *LeEIL-1* in each three lines of WT, EV, Ei, and EO reduced dramatically at 12 h, increased at 1 day in most hairy root lines, constantly declined, and then reached the lowest level at 5 days.

The transcript levels of *LeEIL-1* significantly exhibited varied increase rates in each type of hairy roots cultured in M9 from 5 to 9 days (Fig. 3d). In hairy roots of WT, EV, and Ei, the *LeEIL-1* transcripts presented a rapid increase from 5 to 6 days, and then continually increased for 6–9 days. The *LeEIL-1* transcript level in EO hairy root lines, however, slightly reduced from 6 to 9 days and maintained a relatively high level compared with those in WT, EV, and Ei hairy root lines at 9 days. Consistent with the visual inspection results on shikonin production in M9 medium, the highest *LeEIL-1* transcript level was found in EO lines, the lowest level in Ei lines, and the medium level in EV or WT lines from 5 to 9 days (Fig. 3d). It is obviously, the sixth day in the M9 medium is the important time point at which the expression level of *LeEIL-1* in different hairy roots should be compared. This result indicated that *LeEIL-1* expression might be concurrent with the accumulation of shikonin and its derivatives.

Interestingly, we noticed that *LeEIL-1* was down-regulated at the early stage within 5 days no matter it was located downstream of CaMV-35s promoter or not. In the two-stage culture system, the antibiotic- and hormone-free B5 medium was used as the growth medium for multiplying hairy roots under constant light condition, whereas the hormone-containing M9 medium was employed for hairy roots to produce shikonin and its derivatives under continuous dark culture condition. We speculated that the dramatic differences (medium components and culture condition) between these two stages might affect the expression of *LeEIL-1* at the early stage when transferred *L. erythrorhizon* hairy roots from B5 multiplication medium into M9 production medium.

Since shikonin is biosynthesized in the dark, we also detected the effect of light signal on the expression pattern of *LeEIL-1*. The WT-1 hairy root line was randomly selected and pre-cultured in B5 for 2 days under constant light condition. Similar to the expression pattern of *LeERF-1*, which is another ET response factor reported in our previous study [40], *LeEIL-1* was induced by dark (Fig. 3e). This result was also consistent with the dark-induced mRNA levels of *EIN3* in *Arabidopsis* [41].

### *LeEIL-1* is a positive regulator of shikonin production

**Fig. 4:**
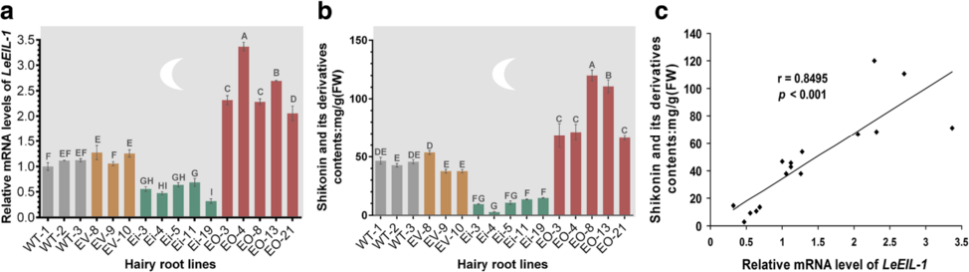
*LeEIL-1*expression levels (**a**) and shikonin production (**b**) of the randomly selected hairy root lines. WT, EV, Ei, and EO hairy root lines cultured in M9 production medium for 6 days. The values are means ± SD (*n* = 3), and the *bars* with different *capital letters* indicate significant differences at *P <* 0.01 (Least Significant Difference). The *deep gray* and *moon logo* indicate the hairy roots were cultured in the dark. Scatter diagram showing the significantly positive linear relationship between *LeEIL-1* expression level and shikonin production (*r* = 0.8495; *P* < 0.001) (**c**)

Based on the above results, we speculated that *LeEIL-1* positively mediates ET-regulated shikonin biosynthesis. To validate the speculation, we performed the relationship analysis between the expression level of *LeEIL-1* (Fig. 4a) and the shikonin contents (Fig. 4b) in various hairy roots in M9 production medium for 6 days. In addition, as for different hairy root types (WT, EV, Ei and EO), only the expression levels of *LeEIL-1* affected by RNAi or overexpresison were different; all other factors, including M9 medium, culture condition (in darkness at 26 °C), were same for them in the research system.

The results showed that both the *LeEIL-1* expression levels and shikonin production were significantly increased in EO hairy root lines, but dramatically declined in Ei in comparison with those in WT or EV control lines (*P* < 0.01) (Fig. 4a and b). Thus, a significantly positive linear correlation between shikonin content and *LeEIL-1* expression level was deduced based on the results of all lines detected above (*r* = 0.8495; *P* < 0.001) (Fig. 4c). Furthermore, a number of high-yield shikonin hairy roots had been obtained from EO (Additional file 5: Figure S5), suggesting the strategy of *LeEIL-1-*overexpression has great potential to obtain hairy roots with high-yield shikonin production.

### *LeEIL-1* confers shikonin production by regulating the expression of key genes for shikonin biosynthesis

As EIN3/EILs act as key positive regulators at the most downstream of ET signaling transduction pathway [39, 42], we deduced that the expression of *LeEIL-1* is possibly associated with the downstream target genes which regulate shikonin formation. Further research on genes involved in ET signaling transduction pathway and shikonin biosynthesis pathway under the mechanism of overexpression and RNAi of *LeEIL-1* will bridge the gap between shikonin production and accumulation of *LeEIL-1* transcripts.

**Fig. 5:**
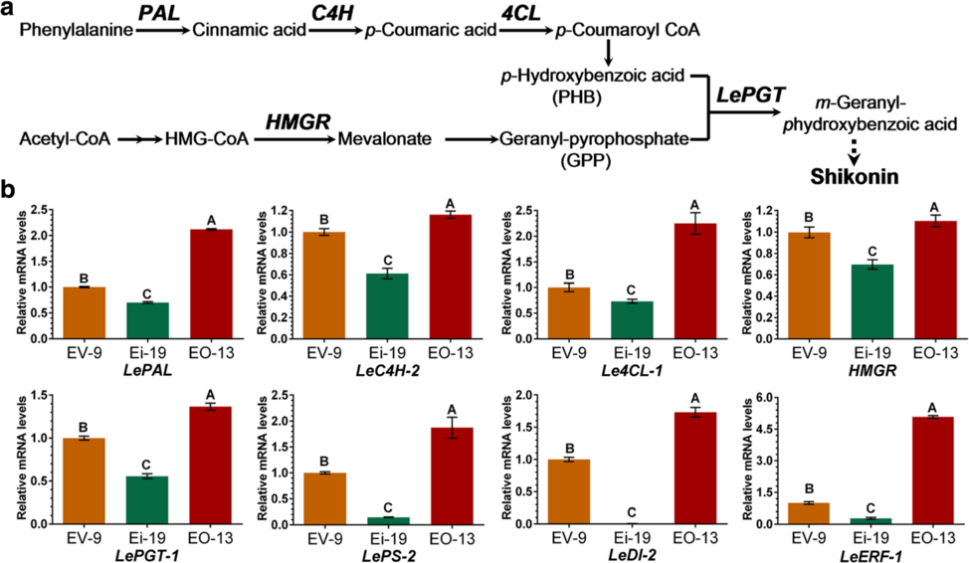
Expression patterns of shikonin biosynthesis-related genes regulated by *LeEIL-1.* (**a**) The metabolic pathway outline for the biosynthesis of shikonin and its derivatives. The genes encoding crucial enzymes of this pathway are marked in *bold* and *italic* fonts; (**b**) Transcript levels of the key shikonin biosynthesis-related gene in EV, Ei, and EO hairy root lines cultured in M9 medium for 6 days. A representative example from two biological experiments is shown; the values are means ± SD (*n* = 3) and the *bars* with different *capital letters* indicate significant differences at *P* < 0.01 (Least Significant Difference)

Since there was no difference for shikonin production and expression level of *LeEIL-1* between EV and WT, and to avoid the transgenic effects of plasmid, three pBI121 vector containing lines (i.e., EV-9, Ei-19, and EO-13) cultured in M9 for 6 days were selected for further analyses of the expression of several key genes implicated in shikonin biosynthesis. These genes include the speculated downstream gene of *LeEIL-1* (*LeERF-1*), the key genes involved in biosynthetic pathway of shikonin (*LePAL*, *LeC4H-2, Le4CL-1*, *HMGR*, *LePGT-1*) (Fig. 5a), and the genes possibly responsible for the transportation, stabilization and/or excretion of shikonin pigments (*LeDI-2* and *LePS-2*).

The expression level of *LeERF-1* was significantly up-regulated in EO-13 but down-regulated in Ei-19 compared with that in the EV-9 control (*P* < 0.01). Similarly, *LeEIL-1* overexpression significantly up-regulated the mRNA accumulation of *LePAL*, *LePGT-1*, *LeC4H-2*, *LePS-2*, *Le4CL-1*, *LeDI-2* and *HMGR* in EO hairy root line (*P* < 0.01). By contrast, RNAi-mediated knockdown of *LeEIL-1* in Ei hairy roots significantly suppressed the expression of all genes, especially *LeDI-2* (*P* < 0.01) (Fig. 5b). We speculated that the up- or down-regulated transcripts of *LeEIL-1* in turn altered the transcription of most genes in the biosynthetic pathway of shikonin. In the hairy roots cultured in M9, expression patterns of these shikonin biosynthesis-related genes (*LePAL*, *LePGT-1*, *LeC4H-2*, *LePS-2*, *Le4CL-1*, *LeDI-2* and *HMGR*) matched to the expression pattern of *LeEIL-1*, as well as shikonin production at the time point of 6 days (Fig. 4a and b). This observation indicated that these shikonin biosynthesis-related genes were induced by *LeEIL-1* and might be involved in the *LeEIL-1*-regulated shikonin production.

*LeDI-2* gene does not participate in the biosynthesis of shikonin, however, it possibly functions in the stabilization and/or transport of shikonin [8, 17]. *LePS-2* is possibly a key gene functioning in the trapping and/or intra-cell wall excretion of shikonin pigments [16]. These studies collectively indicated that the regulation of *LeEIL-1* in shikonin accumulation possibly also includes the event of excretion, stabilization, and/or transportation of shikonin and its derivatives after biosynthesis. Hence, a certain cooperative relationship is necessary between *LeEIL-1* and genes participating in shikonin biosynthesis in *L. erythrorhizon*.

Taken together, *LeEIL-1* confers shikonin production through the regulation of key genes participating in shikonin biosynthesis.

## Discussion

### *LeEIL-1* is a pivotal target gene contributing to efficient shikonin formation

In the signaling network of plant, EIN3/EIL1 acts as a key integration node between ET and other signals [30]. EIN3/EIL1 activates the expression of downstream genes, in which ERFs are the typical ones [39, 43]. ERFs then bind to the GCC box *cis*-elements of many genes regulated by ET, and function positively by activating ET responses [44].

In the medicinal plant *L. erythrorhizon*, shikonin and its derivatives are specifically accumulated in the dark but are suppressed under white or blue light condition. Consistent with the mRNA level of dark-inducible genes *LeERF-1* [40] and *LeDI-2* [17], *LeEIL-1* was also up-regulated in the dark. This finding indicated that light signal acts as a pivotal regulator of *LeEIL-1* during the process of shikonin biosynthesis.

As the first precursor for shikonin biosynthesis, *p*-hydroxybenzoic acid (PHB) is synthesized in the phenylpropanoid pathway. PHB is regulated by sequential enzymes, namely, PAL, C4H, and 4CL [10, 45]. The other precursor is geranyl pyrophosphate (GPP), which is synthesized in the isoprenoid pathway catalyzed by HMGR [12]. The formation of *m*-geranyl-phydroxybenzoic acid (GBA) is the pivotal step of shikonin formation. In this process, the substrates PHB and GPP were catalyzed by LePGT to form GBA, thereby synthesizing shikonin pigmens in *L. erythrorhizon* [15, 46] (Fig. 5a). In this study, *LeEIL-1* overexpression enhanced the expression of the downstream gene of *LeEIL-1* (*LeERF-1*) and a subset of key genes related to shikonin biosynthesis, excretion and/or transportation and stabilization (i.e., *LePAL*, *LePGT-1*, *LeC4H-2*, *LePS-2*, *Le4CL-1*, *LeDI-2* and *HMGR*) (Fig. 5b), and thus increased shikonin accumulation. Conversely, RNAi of *LeEIL-1* effectively decreased shikonin accumulation and down-regulated the above genes. Herein, we speculate that both the isoprenoid pathway and the phenylpropanoid pathway for shikonin biosynthesis were regulated by *LeEIL-1*, but further direct evidences should be provided in future studies. Moreover, the regulatory process also includes the transportation and/or excretion and stabilization of shikonin and its derivatives after formation. In a word, *LeEIL-1* is one of the most important contributors possibly in the ET-regulated shikonin biosynthesis in *L. erythrorhizon*.

**Fig. 6:**
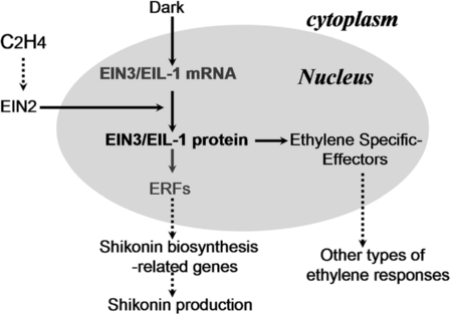
A proposed model illustrates the function of EIN3/EIL-1 in regulating shikonin production. The *dotted lines* represent regulatory steps, in which a direct physical link between upstream and downstream components has yet to be demonstrated

Although the up-regulated transcripts of some EIN3/EIL members contribute to constitutive ET responses, exogenous ET can not affect the EIN3/EIL mRNA levels [47, 48]; nevertheless, the abundance of the EIN3/EIL protein considerably increased upon ET exposure because of the strong stabilization of this protein [42]. The accumulation of EIN3/EIL protein is primarily regulated at post-transcriptional level in vivo, and EIN2 is essential in regulating ET-induced EIN3/EIL1 [49, 50]. Based on the results of previous and present studies, we propose a model for describing the *LeEIL-1*-regulated shikonin production possibly in the ET signaling cascade (Fig. 6); in this model, which is similar to that described by Li et al. (2013), dark-induced *LeEIL-1* acts as a positive regulator in ET-regulated shikonin formation. Therefore, hairy roots with high-yield of shikonin could be obtained by using the *LeEIL-1*-overexpression strategy (Additional file 5: Figure. S5).

### *LeEIL-1* is a possible integration node between ET and other signal molecules

In *Arabidopsis*, EIN3 and its close homolog EIL1 regulate myriad ET responses [39, 47]. In the life cycle of plant, EIN3/EIL1 acts as a regulator of ET signaling as well as myriad processes, such as the development and stress responses [29, 32], plant immunity defenses [28], and crosstalk between ET and jasmonate [30]. Moreover, EIN3/EIL1 is also involved in plant morphological phenotype changes [43], the de-etiolation of seedlings, and leaf senescence [41, 51].

In this study, the polymorphism and growth rate of hairy roots changed under the condition of either *LeEIL-1*-overexpression or *LeEIL-1*-RNAi (Fig. 1b; Additional file 2: Figure. S2). Meanwhile, callusing, differentiation and regeneration phenomena easily occurred in Ei hairy root lines cultured in B5 solid or liquid medium (Fig. 1c and d), compared with that in other hairy root lines cultured under the same condition. Thus, we speculate that *LeEIL-1* possibly regulates other inputs or ET specific effectors in multiple layers in the ET signaling cascade, thereby inducing the cascade of ET responses for cell development, differentiation, and regeneration. It will thus be interesting to investigate whether *LeEIL-1* acts as one of the convergence points between ET and other signal molecules in the complex network of ET transduction pathway in *L. erythrorhizon*.

## Conclusions

Although the biosynthetic pathway of shikonin and its derivatives has been basically well-characterized, the molecular mechanism and key target regulators of shikonin formation are largely unknown. In the present study, by using an excellent two-stage hairy root culture system as the basal condition for shikonin production, we provided evidences for the positive role of *LeEIL-1* in regulating shikonin formation. In hairy root lines of EO, *LeEIL-1*-overexpressing significantly enhanced shikonin content in comparison with that in Ei, as well as the control lines of WT or EV under the same culture conditions. Our findings paved the way for future work aiming at extending our knowledge of the molecular regulatory mechanism of ET in shikonin biosynthesis. Furthermore, we offered a key target gene which has great potential for efficient shikonin biosynthesis in genetic engineering of *L. erythrorhizon*. Future studies focusing on the direct function of *LeEIL-1* or other *LeEILs* in the ET signaling transduction pathway in *L. erythrorhizon* will give more evidences for the regulation of ethylene on shikonin biosynthesis.

## Methods

### Plant materials and treatment

The seeds of *L. erythrorhizon* Sieb. *et* Zucc were collected in the field location of Ulanhot city, Inner Mongolia Autonomous Region of China (N46°04′13.55″, E122°07′42.15″) in accordance with local legislation, and no specific permission was required for this study. Seed stratification and sterilization, as well as the seedling culture condition were essentially as we previously reported [52]. The robust seedlings were used for hairy root induction.

### Plasmid construction and transformation

The plasmid of *LeEIL-1-*overexpression or *LeEIL-1-*RNAi was constructed based on the plant expression vector pBI121-*eGFP*. XbaI/BamHI were selected for the digestion of pBI121-*eGFP* vector, the coding region of *LeEIL-1* (1905 bp) was cloned and introduced into the cleavage site, with the cDNA expression cassette between the CaMV35s promoter and the *eGFP*, thereby generated the plasmid of pBI121-*LeEIL-1*-Overexpression [52].

To construct the pBI121-*LeEIL-1*-RNAi plasmid, a strategy similar to the previous reports was adopted [52–54]. The intron-containing intermediate vector pUCCRNAi was used [55]. A sequence of *LeEIL-1* (348 bases) was selected based on the bioinformatics analyses of *LeEIL-1*. The sense sequence was cloned and inserted into pUCCRNAi in positive orientation, thereby generated a vector of pUCCRNAi-intron-F. The anti-sense sequence was also amplified and inserted into pUCCRNAi-intron-F in reverse orientation. This process generated plasmid of pUCCRNAi-R-intron-F. To obtain the target inverted repeat sequence, the SpeI/XbaI were selected for the digestion of pUCCRNAi-R-intron-F plasmid. Finally, the sequences obtained were inserted in the pBI121-*eGFP* vector, thereby generated the plasmid of pBI121-*LeEIL-1*-RNAi [52, 56].

All plasmids (pBI121-*LeEIL-1*-RNAi, pBI121-*LeEIL-1*-Overexpression and pBI121-*eGFP*) were transformed into the *Escherichia coli* strain TOP10 for amplification and PCR verification. Finally, these recombined expression plasmids were introduced into *A. rhizogenes* strain ATCC15834 as previously reported [52, 57, 58]. All primers for the construction of plasmids and verification of ATCC15834 carrying vectors are listed in Additional file 6: Table S1.

### Induction and cultivation of hairy roots

The hairy root system was used for shikonin biosynthesis and expression analysis of *LeEIL-1* via the two-stage culture system [8, 9]. The infection strains used for hairy root induction were as follows: *A. rhizogenes* strain 15834 (WT), and *A. rhizogenes* 15834 strain carrying the plasmid pBI121-*eGFP* (EV), pBI121-*LeEIL-1-*Overexpression (EO) or pBI121-*LeEIL-1*-RNAi (Ei). All stains were inoculated in YEB liquid medium in a rotary shaker (120 rpm, 26–28 °C) for about 36 h. Kanamycin (50 mg l^−1^) was added to the YEB medium for antibiotic resistance screening of EV, Ei and EO. When the OD_600_ of infection strains reached 0.5, acetosyringone (AS) at 100 μM was supplemented.

Hairy roots were then induced with the method as we previously reported [36, 52]. In brief, seedling nodes were pricked with a needle soaked in the infection medium. Then the vaccinated seedlings were transplanted to MS solid medium under subdued light or dark condition till the hairy roots appeared after 2 weeks, and over 15 lines for each type of hairy root were generated. At the infection sites, individual hairy root of about 1 cm was excised. After the elimination of residual *Agrobacterium* with cefotaxime*,* hairy roots were cultured in B5 solid medium (antibiotic- and hormone-free). The conditions of stock cultivation were set as follow: under subdued light at 26–28 °C.

The two-stage culture system was adopted for hairy root cultivation as previously described [7–9, 21]. Hairy roots cultured on B5 solid medium were cut, and then transferred into B5 multiplication liquid medium (antibiotic- and hormone-free) for fast amplification under continuous white light at 26–28 °C with a constant shaking rate of 80 rpm. Hairy roots were subcultured every 15 days (Fig. 3b).

For shikonin biosynthesis, hairy roots were transferred from B5 multiplication medium into M9 production medium and cultured in the dark at 26–28 °C with a constant shaking rate of 80 rpm [21, 36, 52, 59] (Fig. 3c).

### Subcellular localization analysis of LeEIL-1

The confocal laser scanning fluorescence microscope (LSFM, FV10-ASW, Olympus, Japan) was used for the detection of eGFP fluorescence. Excitation and emission wavelength were 488 and 510 nm, respectively [39, 60].

### Real-time PCR analysis

Hairy root lines cultured in B5 multiplication medium or in M9 production medium were selected for mRNA level analysis. TRIzol reagent (TaKaRa Biotech, Shiga, Japan) was used to extract total RNA from hairy roots. First-strand cDNA was synthesized by using 1 μg of the total RNA and M-MLV reverse transcriptase (Promega, Madison, WI, USA). The real-time PCR was performed by using CFX96TM (Bio-Rad, USA) and the SYBR Green real-time PCR Master Mix (Toybo Co., Ltd., Osaka, Japan). Primers for real-time PCR analysis were designed based on the sequences in the GenBank database [24] (Additional file 7: Table S2). The glyceraldehyde-3-phosphate dehydrogenase gene (*GAPDH*), an internal reference gene, was selected as in our previous reports [24, 36, 52]. The thermal program was performed using the following parameters: denatured at 95 °C for 1 min, followed by 40 amplification cycles (95 °C for 15 s, 55 °C for 15 s, 72 °C for 45 s). Melting curves were performed after 40 cycles to confirm the specificity of the reactions. Relative expression level was calculated using the ΔΔCt method [24, 35, 36, 40, 52]. At least two to three biological replicates and three technical replicates were performed for the data analysis.

### Measurement of shikonin contents

Measurement of shikonin contents was conducted as we previously reported [19, 20, 24, 36, 52]. In brief, shikonin and its derivatives were extracted with petrol ether and measured by using the WFZ UV-2800H spectrophotometer (Unico, Shanghai, China). Absorption of these pigments was detected at the characteristic wavelength of 520 nm. Shikonin contents were determined using a standard curve as we previously reported (shikonin content = 41.66 × OD_520_ × dilution fold) [19, 20, 24, 52]. The total shikonin content including the pigments extracted from both the hairy roots and the M9 production medium was expressed as mg/g fresh weight (FW) of hairy root.

### Abbreviations

ET, ethylene; eGFP, enhanced green fluorescent protein; EO, pBI121-*LeEIL-1*-Overexpression; EIN3/EIL, Ethylene-insensitive 3/EIN3-like protein; Ei, pBI121-*LeEIL-1*-RNAi; EV, pBI121-*eGFP* Empty Vector; *LeEIL-1*, *L. erythrorhizon* EIN3-like protein gene 1; *LePAL*, *L. erythrorhizon* phenylalanine ammonia-lyase gene; *LeC4H-2*, *L. erythrorhizon* cinnamic acid 4-hydroxylase gene 2; *Le4CL-1*, *L. erythrorhizon* 4-coumaric acid:CoA ligase gene 1; *HMGR*, 3-hydroxy-3-methylglutaryl-coenzyme A reductase gene; *LePGT-1*, *L. erythrorhizon p*–hydroxybenzoate:geranyltransferase gene 1; *LePS-2*, (*L. erythrorhizon* pigment callus-specific gene 2); *LeDI-2*, *L. erythrorhizon* dark-inducible gene 2; *LeERF-1*, *L. erythrorhizon* ethylene response factor gene 1; RNAi, RNA interference; WT, wild type.

## Additional files

Additional file 1: Figure S1. Verification of the constructed recombinant vectors. (A) Identification of the *eGFP* gene in the pBI121-eGFP vector by using the primer pair eGFP-F/R; (B) Amplification of the inserted target sequence in the pBI121-*LeEIL-1*-RNAi vector by using the primer pair 35S-F/GFP-R. (C) Amplification of the inserted target sequence of *LeEIL-1* in the pBI121-*LeEIL-1*-Overexpression vector by using the primer pair 35S-F/GFP-R. Primer sets are listed in Additional file 6: Table S1. (TIF 456 kb) Additional file 2: Figure S2. Stock culture of WT (A), EV (B), Ei (C) and EO (D) hairy roots of *L. erythrorhizon* on B5 solid medium (antibiotics-free and hormone-free). (TIF 3277 kb) Additional file 3: Figure S3. The time course of fresh weight (FW) of WT-1, EV-9, Ei-19 and EO-13 cultured in B5 multiplication medium (continuous light at 26–28 °C with a constant shaking rate of 80 rpm). The inoculum size of 0.2 g hairy roots was subcultured in 20 ml medium/50 ml flask. Three biological replicates were performed; the value of Ei-19 or EO-13 is significantly different from that control line of WT-1 or EV-9 at each time point from 3 to 15 days, respectively (Student’s *t*-test, *P* < 0.05). (TIF 83 kb) Additional file 4: Figure S4. Time-course accumulation of shikonin in four typical hairy root lines. Value of Ei-19 or EO-13 is significantly different from that of the control line WT-1 or EV-9 at each time point from 3 to 12 days, respectively (Student’s *t*-test, *P* < 0.05). (TIF 125 kb) Additional file 5: Figure S5. Comparison of growth characteristics between the typical low-yield shikonin line (A) of Ei and high-yield shikonin line (B) of EO. Hairy roots were cultured in M9 production medium in the dark for 6 days (20 ml medium/50 ml flask). (TIF 3133 kb) Additional file 6: Table S1. Primers for *LeEIL-1* cDNA cloning and vector construction and verification. (DOC 39 kb) Additional file 7: Table S2. Real-time PCR primers for gene expression analysis. (DOC 34 kb)
