# Supplementary figures and images for: Transgenic studies reveal the positive role of LeEIL-1 in regulating shikonin biosynthesis in Lithospermum erythrorhizon hairy roots
Source: BMC Plant Biol. 2016 May 26;16:121. doi: 10.1186/s12870-016-0812-6 (PMC4880835; doi:10.1186/s12870-016-0812-6)

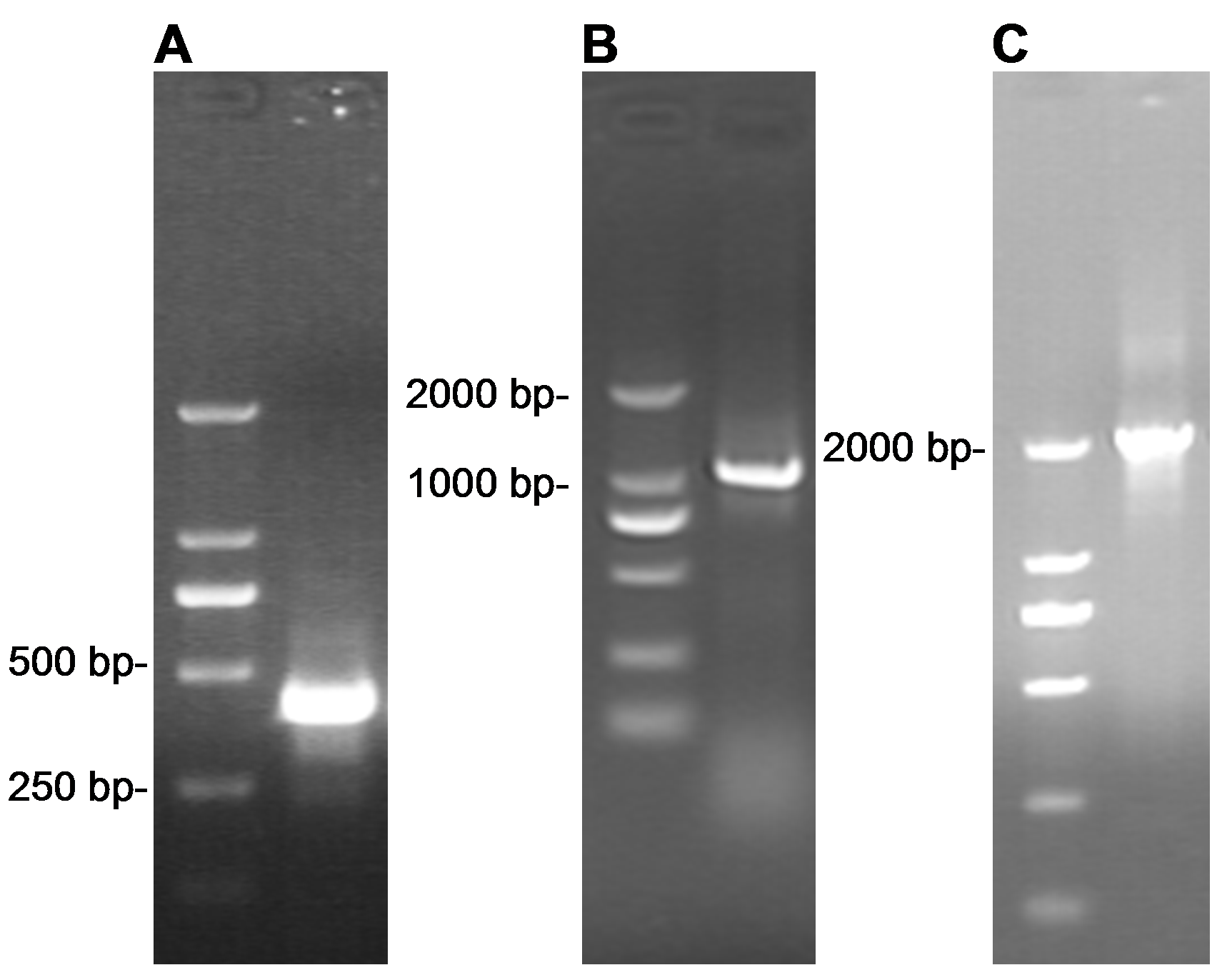

Supplement: Additional file 1: Figure S1. — Verification of the constructed recombinant vectors. (A) Identification of the eGFP gene in the pBI121-eGFP vector by using the primer pair eGFP-F/R; (B) Amplification of the inserted target sequence in the pBI121-LeEIL-1-RNAi vector by using the primer pair 35S-F/GFP-R. (C) Amplification of the inserted target sequence of LeEIL-1 in the pBI121-LeEIL-1-Overexpression vector by using the primer pair 35S-F/GFP-R. Primer sets are listed in Additional file 6: Table S1. (TIF 456 kb) [file 12870_2016_812_MOESM1_ESM.tif]

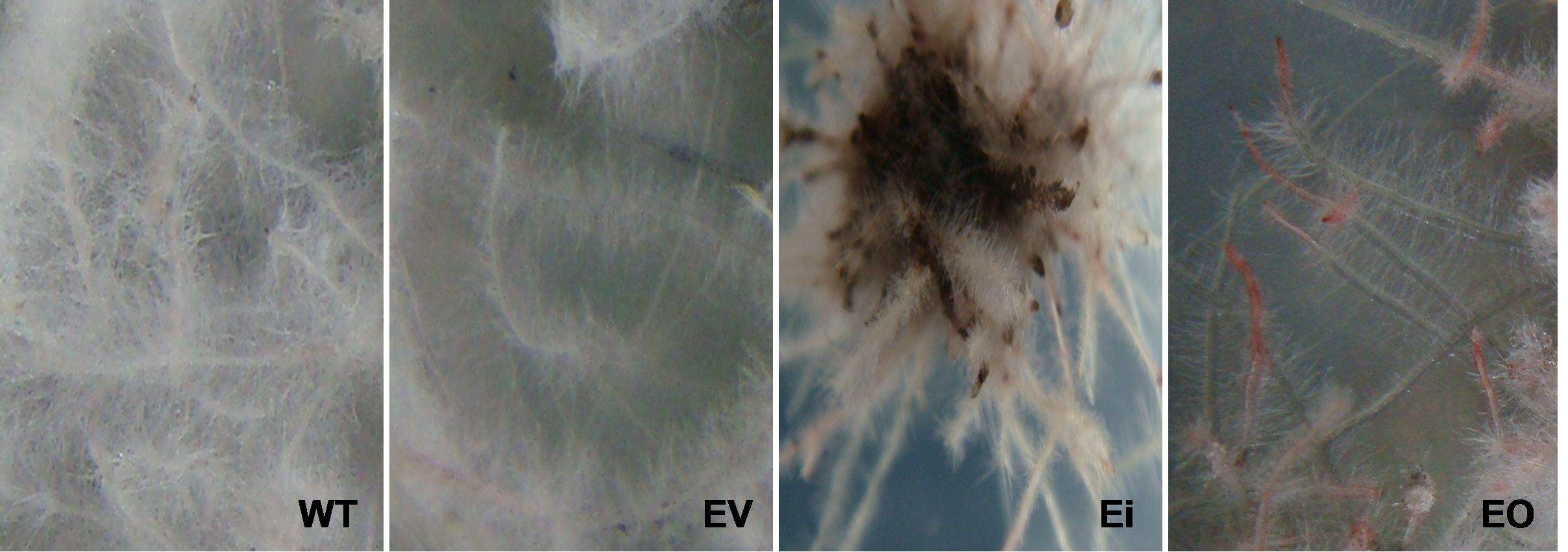

Supplement: Additional file 2: Figure S2. — Stock culture of WT (A), EV (B), Ei (C) and EO (D) hairy roots of L. erythrorhizon on B5 solid medium (antibiotics-free and hormone-free). (TIF 3277 kb) [file 12870_2016_812_MOESM2_ESM.tif]

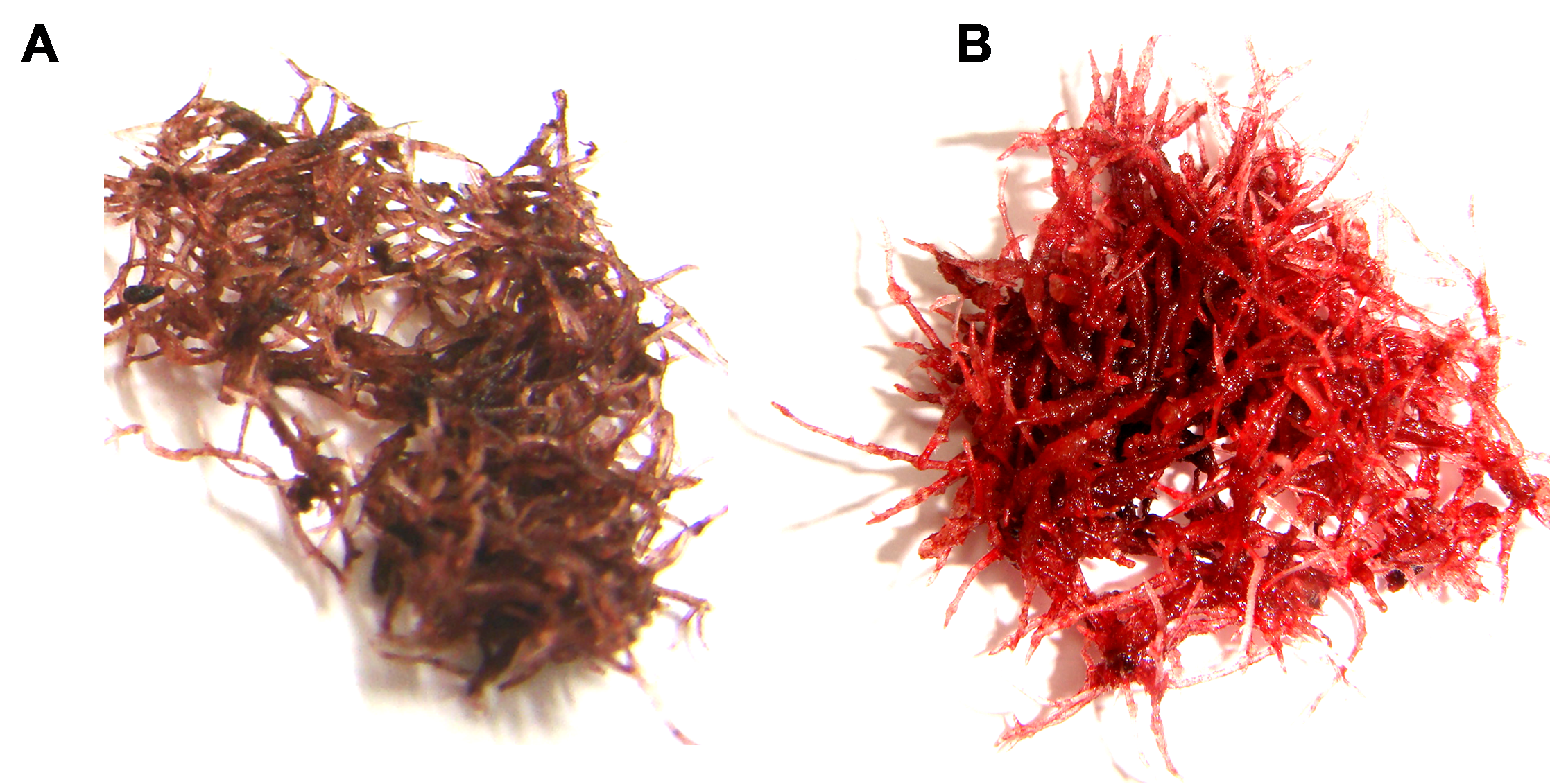

Supplement: Additional file 5: Figure S5. — Comparison of growth characteristics between the typical low-yield shikonin line (A) of Ei and high-yield shikonin line (B) of EO. Hairy roots were cultured in M9 production medium in the dark for 6 days (20 ml medium/50 ml flask). (TIF 3133 kb) [file 12870_2016_812_MOESM5_ESM.tif]
