# Supplementary material for: Transgenic studies reveal the positive role of LeEIL-1 in regulating shikonin biosynthesis in Lithospermum erythrorhizon hairy roots
Source: BMC Plant Biol. 2016 May 26;16:121. doi: 10.1186/s12870-016-0812-6 (PMC4880835; doi:10.1186/s12870-016-0812-6)
Supplement: Additional file 6: Table S1. — Primers for LeEIL-1 cDNA cloning and vector construction and verification. (DOC 39 kb) [file 12870_2016_812_MOESM6_ESM.doc]

**Additional file 6:**

**Table S1.** Primers for *LeEIL-1* cDNA cloning and vector construction and verification.

| **Primer type** | **Size of target sequence (bp)** | **Primer name** | **Sequence (5’→3’)** |
| --- | --- | --- | --- |
| Cloning of coding region of *LeEIL-1* and construction of  *LeEIL-1*-overexpression vector | 1905 | LeEIL-1-OE-F | CTAGTCTAGAATGATGATGTTCGAGGAGATGGGGT |
|  |  | LeEIL-1-OE-R | CTAGTCTAGAAAACCAGATGGATTCGTCCTGCTTT |
| Intron cloning for the construction of *LeEIL-1*-RNAi vector | 213 | Intron-F | GGTACGGACCGTACTACTCTATTCGTT |
|  |  | Intron-R | CCTATATAATTTAAGTGGAAAAAAAGGTTAAC |
| Sense sequence cloning for the construction of *LeEIL-1*-RNAi vector | 348 | LeEIL-1-RNAi-SF | CATGGATCC CCTCATACCTTGCAAGAGCTTCAAGACA |
|  |  | LeEIL-1-RNAi-SR | CATTCTAGA TGCCAGCCATGTCGCACTCTCCTTCGCT |
| Anti-sense sequence cloning for the construction of *LeEIL-1*-RNAi vector | 348 | LeEIL-1-RNAi-AF | CATACTAGTTGCCAGCCATGTCGCACTCTCCTTCGCT |
|  |  | LeEIL-1-RNAi-AR | CATAGATCT CCTCATACCTTGCAAGAGCTTCAAGACA |
| Verification of the target sequence in vectors of pBI121-*LeEIL-eGFP*/pBI121*-LeEIL*-1-RNAi | 2072/1076 | 35S-F | CACTATCCTTCGCAAGACCCT |
|  |  | GFP-R | GCTGAACTTGTGGCCGTTT |
| Verification of *Agrobacterium rhizogenes* ATCC15834 and hairy roots | 626 | rolC-F | CTCCTGACATCAAACTCGTC |
|  |  | rolC-R | TGCTTCGAGTTATGGGTACA |
| Verification of *E. coli* TOP10 or *Agrobacterium rhizogenes* ATCC15834 harboring the constructed vectors | 319 | eGFP-F | ACCCTCGTGACCACCCTGAC |
|  |  | eGFP-R | AGTTCACCTTGATGCCGTTC |
