# Supplementary material for: Transgenic studies reveal the positive role of LeEIL-1 in regulating shikonin biosynthesis in Lithospermum erythrorhizon hairy roots
Source: BMC Plant Biol. 2016 May 26;16:121. doi: 10.1186/s12870-016-0812-6 (PMC4880835; doi:10.1186/s12870-016-0812-6)
Supplement: Additional file 7: Table S2. — Real-time PCR primers for gene expression analysis. (DOC 34 kb) [file 12870_2016_812_MOESM7_ESM.doc]

**Additional file 7:**

**Table S2.** Real-time PCR primers for gene expression analysis.

| **Real-time PCR primer type** | **Gene name** | **GenBank Accession number** | **Sense primer** (5’→3’) | **Anti-sense primer** (5’→3’) |
| --- | --- | --- | --- | --- |
| For ethylene signaling pathway related genes | *LeEIL-1* | FJ890314 | GCTCCTCAGGATGAACCAG | TCCTTACCCTTGCTGCTCATCTC |
|  | *LeERF-1* | GQ246186.1 | GCCAATTCATTCCATCGC | GTCCCAAATGTTCCAAGC |
| For shikonin biosynthesis pathway- related genes | *LePAL* | D83075.1 | GCAGCCATGCTTGTTAGGA | GGTGTTGAGGAACTTGGTGATA |
|  | *LeC4H-2* | AB055508.1 | AGGGTGAAATCAACGAGG | ATACGGAGGCGAAGGGTC |
|  | *Le4CL-1* | D49366.1 | CCTATTCGTCAGGGACTACAG | GCACATCACAACATCTTCGT |
|  | *HMGR* | X74783.1 | TTGGAGTTCCTACAGAATGAGT | GCGGCTGGTTTCTTGTCA |
|  | *LePGT-1* | AB055078.1 | CTCTTAGGCTCCTCTGCT | CGTCGTCCACCTTATCTT |
|  | *LeDI-2* | D45901.1 | TAATCTCGTTACGGCTCAA | GCAAAGGCAAGCACCAAC |
|  | *LePS-2* | AB047916.1 | ATCAACGGAACGGACCAAGT | TTGAAGGCATAACGGACACC |
